# From Waste to Consumption: Tomato Peel Flour in Hamburger Patty Production

**DOI:** 10.3390/foods13142218

**Published:** 2024-07-15

**Authors:** Betül Karslıoğlu, Eda Demirok Soncu, Beyzanur Nekoyu, Erdem Karakuş, Gülsedef Bekdemir, Barış Şahin

**Affiliations:** 1 Department of Gastronomy and Culinary Arts, Faculty of Tourism, Hasan Kalyoncu University, Gaziantep 27000, Turkeygulsedefbekdemir@gmail.com (G.B.);; 2 Department of Food Engineering, Faculty of Engineering, Ankara University, Ankara 06110, Turkey; edemirok@eng.ankara.edu.tr

**Keywords:** tomato peel flour, food waste, food sustainability, meat products, hamburger patty

## Abstract

Tomato is a widely cultivated crop and its processing produces large quantities of wastes, such as pulp, seed, and peel. In recent years, the valorization of these wastes in the production of high-value-added food products has gained popularity in achieving environmental sustainability and zero waste. From this viewpoint, dried tomato peel (DTP-1%, 2%, 3%, 4%) flour was included in hamburger formulations. In patty samples, ash, carbohydrate, and dietary fiber amounts were increased due to the high fiber content of DTP flour, while moisture and fat percentages decreased with increasing amounts of DTP flour (*p* < 0.05). The inclusion of DTP flour retarded lipid oxidation during cooking (*p* < 0.05). The significantly highest cooking yield was calculated in samples including 4% DTP flour. In parallel, water-holding capacity, moisture, and fat retention values increased with increasing levels of DTP flour (*p* < 0.05). The enrichment of patties with DTP flour resulted in hard texture, less gumminess, and a darker, more reddish and yellowish color (*p* < 0.05). Hamburger samples containing 1% or 2% DTP flour were graded with closer scores in the sensory panel as compared to the control (0% DTP). Overall, our findings demonstrated that DTP flour up to 2% could be used to improve the nutritional and technological properties of patty samples.

## 1. Introduction

Waste management is one of the most critical concerns in the food sector, and as food safety and sustainability become more important, subjects such as recycling food waste and manufacturing high-value-added goods have gained attraction [1,2,3]. While annually increasing food waste has been reported to be approximately 1.3 billion tons globally, according to the United Nations (UN) Food Waste Index Report 2021, the per capita annual food waste in Turkey has been determined to be 93 kg per person [4,5]. The fruit and vegetable sector produces the highest amount of waste in Turkey, with tomatoes being the most manufactured agricultural product [5]. The industrial processing of tomatoes yields large quantities of by-products that contain a variety of reusable and high-value components with economic potential [6,7].

Tomatoes are consumed both fresh and in various processed forms, with the majority being made into products such as paste, sauce, puree, soup, juice, ketchup, and canned tomatoes [8,9]. Due to the high demand, the tomato industry generates approximately 15 million tons of waste both before and after processing. Pre-processing waste, which accounts for about 1–6% of total tomato production, includes damaged and green tomatoes.

The primary post-processing waste is tomato pomace, which consists of a mix of seeds, peels, fibrous parts, and a small amount of pulp [10]. The utilization of these wastes is quite limited [11,12,13]. Skins and seeds comprise about 1–4% (*w*/*w*) of the total tomato processed for tomato products and are usually destined for landfill or to be used for animal feed [14]. While these waste materials contribute to food loss and waste, they also result in environmental pollution due to unnecessary CO_2_ emissions [7]. However, these tomato waste parts contain many valuable components that are beneficial to human health [15]. For instance, tomato peels provide a rich and natural source of dietary fiber and other components [16]. Additionally, numerous studies have highlighted that waste components like tomato seeds and peels are rich in compounds such as dietary fiber, protein, fat, minerals, and carotenoids [14,17,18]. Therefore, the recycling of these wastes offers significant opportunities for developing innovative and nutritious additives to enhance healthy meat products [19,20,21].

Fiber constitutes the major component of tomato peel and serves as a valuable source of human nutrition [22]. Tomato peels, which contain approximately five times more dietary fiber than tomato pulp, contain approximately 8.9% soluble dietary fiber and 48.5% insoluble dietary fiber [23,24]. Direct incorporation of dietary fiber obtained from tomato peels into meat products not only enables the reuse of tomato peels but also enhances the nutritional quality of the final product, thanks to its high fiber content. Considering these properties, the utilization of tomato peels in the formulation of meat patties appears to be a promising approach to improve both the organoleptic and nutritional qualities of the products. Several researchers have studied the addition of dietary fibers to meat products during storage to preserve their features or improve health benefits [17,25,26,27,28,29]. 

However, relatively few researchers have investigated the incorporation of tomato peels to enhance dietary fiber content in meat products. Dietary fiber, a form of carbohydrate, is an important component of nutraceuticals that protects gastrointestinal health [30]. Dietary fiber consumption has been associated with various health benefits, such as the preservation of intestinal integrity and the reduction and regulation of low-density lipoprotein cholesterol and blood sugar levels [31,32,33]. Furthermore, it has been demonstrated that including dietary fiber in specific food formulations improves their nutritional value and sensory appeal while lowering the cost of producing meat products [34,35,36,37]. It is possible to successfully add dietary fiber to a wide variety of foods, including meat products. To create meat products with acceptable sensory qualities in this situation, ingredients like beets, wheat, oats, soy fiber, pea fiber, flaxseed flour, and fruit fibers have been utilized [38]. An alternative ingredient that lowers formulation costs, minimizes cooking loss, and enhances the textural qualities of meat products is fiber from tomato peel [39]. The aim of this study is to investigate the effect of tomato peel waste on the physicochemical, sensory, and textural properties of hamburger patties and to reveal their potential for use. Additionally, this study aims to determine the appropriate DTP (dried tomato peel) flour concentration that can be used in hamburger patties. 

## 2. Materials and Methods

### 2.1. Materials and Reagents

In this study, approximately 12 kg of medium fat (20%) minced meat obtained from mature cattle carcasses (Native Black Cattle) was provided by Aydiz Co. in Gaziantep, Turkey. The research materials included a variety of tomatoes (*Solanum lycopersicum* L.), which were purchased from a local supermarket in Gaziantep. Garlic, onion powder, black pepper, and breadcrumbs to be added to the hamburger patties were supplied from Bağdat Baharat Co. (Ankara, Turkey), in sealed packages. All solvents used in the study were of analytical grade and were purchased from Merck (Darmstadt, Germany) and Sigma-Aldrich (Steinheim, Germany). Protease, 2-Thiobarbituric acid, α-Amylase, Amyloglucosidase, 2 (N-morpholino) ethanesulfonic acid (MES), Tris (hydroxymethyl) aminomethane reagents were obtained from Sigma-Aldrich Merck Group company (St. Louis, MO, USA).

### 2.2. Preparation of Dried Tomato Peel (DTP) Flour

Tomato peel flour was prepared using the method outlined by Savadkooh et al. [40]. After washing the tomatoes with tap water, the tomato peels were removed to a thickness of 2 to 4 mm using a knife. Tomato peels were dried (Magic Mill Pro Food Dehydrator Machine, Upper Saddle River, NJ, USA) at 50 °C for 18 h to a moisture content of 6.2% (wet-weight). The airflow rate was set at 2.5 m/s. The dried tomato peels were ground using a kitchen grinder (Bosch MultiTalent, 3 MCM3501M, Rosenau, Germany). After sifting through a 75-mesh sieve (212 µm), the resulting flour was stored under dark conditions (25 °C) in vacuum-sealed polyethylene bags (Multilayer Thick Type 20 × 26 cm; Klikal, Turkey) until the analysis day. 

### 2.3. Physicochemical Analysis of DTP Flour

The analysis of the proximate composition of DTP flour samples was determined following the procedures outlined in the AOAC methods. Total (TDF), soluble (SDF), and insoluble (IDF) dietary fiber in DTP flour were determined by enzymatic and gravimetric methods described in AOAC (991.43). The titratable acid (TA) as citric acid was determined according to the method of Lisiewska and Kmiecik [41]. A digital pH meter was used to measure the pH of homogenates of flour samples (10 g) in 100 mL of distilled water (Hanna HI 221, Ann Arbor, MI, USA). To determine the physicochemical properties of DTP flour, oil absorption capacity (OAC), water absorption capacity (WAC), and swelling capacity (SC) were analyzed using the methods defined by Okaka and Potter, as well as by Öztürk and Turhan [42,43]. 

### 2.4. Production of Hamburger Patties

Based on the sensory panel results from preliminary trials of hamburger patties and literature outputs, five different concentrations (0%, 1%, 2%, 3%, 4%) of DTP flour were used in hamburger patty formulations, which were created using the recipe described in Table 1. The minced meat was mixed with salt, bread crumbs, water, DTP flour, black pepper, onion powder, and garlic powder. DTP flour was added in its dry form without any pre-treatment. The control group did not include any DTP flour. The hamburger patties were produced in the Culinary Arts Kitchen of the Department of Gastronomy at Hasan Kalyoncu University in Turkey. Within the scope of the study, 1 kg of each batch mixture was produced. For every batch of hamburger mixture, the ingredients were combined using a Kitchen Aid mixer (Bosch MultiTalent, 3 MCM3501M) for 4 min, and the mixture was subsequently divided into five equal portions. 

**Figure 1:**
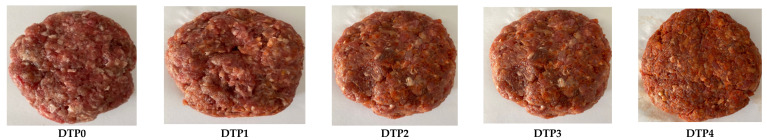
Visual of meatballs including DTP flour at different concentrations: DTP0: Control (without DTP flour), DTP1: Burger including 1% DTP flour, DTP2: Burger including 2% DTP flour, DTP3: Burger including 3% DTP flour, DTP4: Burger including 4% DTP flour.

The first portion was kept as a control. Four different amounts of DTP flour (1%, 2%, 3%, 4%) were added to the remaining four meat portions, respectively, and each group was mixed until a homogeneous distribution was achieved, utilizing the Kitchen Aid mixer (Bosch Multi Talent, 3 MCM3501M, Blaichach, Germany). The meat doughs were left for approximately 30 min at 7 °C before the meatballs were formed. Subsequently, the hamburger patties were shaped to be 50 g ± 0.5 g in weight, with a diameter of 10 cm and a height of 1 cm. The appearance of raw hamburger patties at the end of production is presented in Figure 1. The hamburger patties were pan-fried at 180 °C until they reached a core temperature of 72 °C. All batches were manufactured in two replications at different times. The meat utilized in these formulations had an initial chemical composition of 69.05% moisture, 18.37% protein, and 19.37% fat (wet-basis).

### 2.5. Cooking Procedure and Measurements in Hamburger Patties

After measuring the diameter and thickness of the raw hamburger patties with the help of a digital micrometer (Insize 3109-100A), the meatballs were cooked for 4 min so that the internal temperature of the meatballs was 72 °C. The diameter, thickness, and weight measurements of cooked hamburger samples were taken from five different points. The changes in the cooking yield, thickness, and diameter of the hamburger patties were calculated according to the equations provided by Soncu et al. [33] and Akcan et al. [34]. Moisture and oil retention were also calculated using relevant equations to evaluate fiber quality [44]. Each hamburger sample was wrapped in stretch film, frozen in the refrigerator, and stored at −18 °C until the relevant analyses were completed. Sensory analysis, texture profile analysis (TPA), color, TBARS, and pH measurements were conducted on the same day as the hamburger production.

### 2.6. Physicochemical Parameters and Chemicals Composition of Hamburger Patties

The moisture (950.46), fat (991.36), protein (955.04), a_w_ (978.18), ash (920.153), and dietary fiber contents (991.43) of the samples were analyzed following the methods outlined in AOAC (2000). The pH values of hamburger patties (10 g) were determined by measuring them in 100 mL of distilled water using a digital pH meter (Hanna HI 221, Ann Arbor, MI, USA). Additionally, the carbohydrate content of the samples was calculated by subtracting the sum of moisture, ash, protein, and fat percentages from 100 [45]. Protein, fat, ash, carbohydrate, and dietary fiber results were calculated as g/100 g dry matter (DM). The following factors were also used to evaluate the energy content of hamburger patties: 4 kcal/g for carbohydrates, 9 kcal/g for lipids, and 4 kcal/g for proteins. TBARS values (mg malondialdehyde/kg) for both raw and cooked hamburger patties on day 0 were determined following the method described by Mielnik et al. [46]. In addition to all these parameters, the water-holding capacity of the hamburger samples was determined according to the method provided by Akcan et al. [34].

#### 2.6.1. Color Measurement

The surface color of the hamburger patty samples was assessed at 10 different points using a Minolta Colorimeter (CR 300, Osaka, Japan). The CIELAB L*, a*, and b* values were recorded to indicate lightness, redness (+ for red; − for green), and yellowness (+ for yellow; − for blue), respectively. The color difference (ΔE) for each DTP-including group was computed using the AMSA formula, with the control sample serving as a blank [47].

ΔE = [(L^*^ − *L*_0_^*^)^2^ + (a^*^ − a_0_^*^)^2^ + (b^*^ − b_0_^*^)^2^]^0.5^ (1)

#### 2.6.2. Texture Profile Analysis (TPA)

The textural properties of the cooked hamburger patties were evaluated using a Texture Analyzer (TA Plus, Stable Micro Systems Ltd., Surrey, UK) equipped with a 50 mm aluminum cylindrical probe (model P/50R) and a 50 kg load cell [48]. The pre-test speed was considered to be 1 mm/s with 5 mm/s of test and post-test speed. Hamburger patties were removed from the refrigerator and allowed to sit at room temperature for 1 h. Texture measurements were performed on 5 different meat cut surfaces with a dimension of 1 × 2 × 2 cm per replication. The hardness, springiness, cohesiveness, and chewiness properties of the samples that were double-compressed with a compression ratio of 60% were calculated from the curves.

#### 2.6.3. Sensory Evaluation

Sensory evaluation was performed in a well-lit and well-ventilated room (21 ± 1 °C, 55% ± 5% relative humidity) designed with separated tables at Hasan Kalyoncu University. Before the panel, panelists were informed about the study and signed the consent form. Patties from each formulation were cooked under the previously specified conditions and kept warm in the oven (30 °C) until they were tested. In the sensory panel, hamburger patty samples were numbered with 3-digit random numbers, and unsalted bread and water were provided to panelists to cleanse the palate. A 9-point hedonic scale (1 = extremely dislike, 5 = ‘neither like nor dislike’, and 9 = extremely like) was used to evaluate product liking and acceptability of color, taste, texture, appearance, and general acceptability parameters of cooked hamburger patty samples [49]. Sensory analysis was carried out by experienced panelists aged 25–43, consisting of eight men and 10 women.

### 2.7. Statistical Analysis

Two replications were conducted at different times, and all parameters were measured in duplicate. The mean values of each parameter analyzed in the hamburger patties were evaluated by variance analysis using Minitab statistical software (Version 22.1, Minitab Inc., Enterprise Drive, State College, PA, USA). Regarding TBARS value, two-way ANOVA statistical design was used in which different amounts of DTP flour and cooking treatments were used as independent variables. For the statistical evaluation of the remaining dependent variables, a one-way ANOVA was conducted, with the different amounts of DTP flour as the only independent variable. When the *p*-value was lower than 0.05, the main or interaction effect of variables on results was determined by using Tukey’s post hoc test. The results were given as mean ± SEM (standard error of the mean). On the other side, Principal component analysis (PCA) was also utilized to examine the relationship among hamburger patties (DTP0, DTP1, DTP2, DTP3, DTP4), and Pearson correlation analysis was performed in order to evaluate the relationship between dependent variables by using Minitab Statistical Software (Version 22.1, Minitab Inc., Enterprise Drive, State College, PA, USA).

## 3. Results and Discussion

### 3.1. Physicochemical, Chemical, and Functional Properties of DTP Flour

Table 2 shows some chemical and physicochemical characteristics of DTP flour. The data obtained in the study indicated that tomato peel powder was a rich source of total fiber (72.88%) and carbohydrates (76.58%), with low mass fractions of protein (10.15%), moisture (6.20%), fat (1.61%), and ash (5.46%). Additionally, the pH, titratable acidity, and water activity of the DTP flour were determined to be 4.18, 7.75%, and 0.41, respectively. Similar to our investigation, Grassino et al. found that the pH values ranged from 4.35 to 5.63, ash content was in the range of 1.90% to 3.00%, and total dietary fiber varied between 57.70% and 66.30%, in two different varieties of dried tomato peels [16]. There are very similar studies specifically reporting the composition of tomato by-products, especially tomato peels [16,27,50,51]. 

The data obtained in this study are consistent with some previous studies, while they show significant differences in certain aspects. Gonzalez et al. found that tomato peels have higher protein (13.3%), higher fat (6%), and lower ash content (3%) [51]. Similarly, Elbadrawy et al. reported findings of 78.56% carbohydrates, 10.5% protein, 5.90% ash, and 4.04% fat in tomato peels, which were consistent with our study [27]. The properties of dried tomato peels were found to vary depending on the growing area, climatic conditions, and genetic diversity. Another factor contributing to this variability is the difference in the chemical composition of the seed and peel parts.

The composition of soluble and insoluble fiber fractions in DTP are the main factors affecting the technological functionality and nutritional value of food, and these two parameters (12.13% and 60.75%) are shown in Table 2. According to these results, it was observed that DTP has an extremely high dietary fiber content. This indicates the potential for using tomato peels, which emerge as waste, to develop functional hamburger patties rich in dietary fiber. On average, these values were consistent with the reported results [27,29,52,53]. 

Additionally, in order to determine the physicochemical properties of this DTP rich in high IDF content, water absorption capacity (WAC), oil absorption capacity (OAC), and swelling capacity (SC) were also determined. The length of the fiber structure of DTP flour, the fiber’s particle size, and the condition of the water-soluble fibers were assessed using OAC, WAC, and SC parameters. The results showed that these values were 82.01%, 50.75%, and 140%, respectively [54,55]. Akubor and Owuse also reported similar values in DTP flour [56]. As indicated in Table 2, DTP flour exhibited a higher oil absorption capacity compared to its water absorption capacity. The soluble and insoluble fiber ratio affects the water absorption capacity of the fiber source; hence, DTP flour with a higher proportion of insoluble fiber showed lower water absorption capacity. This phenomenon has been explained by the greater affinity of the non-polar chains of proteins in DTP flour for lipid molecules [57]. Swelling capacity, which is related to water retention capacity, was found to be 140% for DTP. In summary, the tomato peel flour (DTP) analyzed in this study has a high dietary fiber content, consisting largely of insoluble fibers. Due to its chemical and physicochemical properties, DTP has the potential to be a new non-meat ingredient for the preparation of low-calorie, high-fiber meat products. These characteristics indicate that DTP could significantly contribute to the development of healthy and functional products in the food industry. Additionally, the use of DTP can enhance environmental sustainability by reducing food waste and increasing economic value, thereby providing solutions in line with the principles of industrial ecology.

### 3.2. Chemical Composition of Hamburger Patty Samples

The chemical composition, a_w_, and pH value results of cooked hamburger patties with different proportions of DTP flour are summarized in Table 3. As observed, the addition of DTP flour significantly affected all composition parameters (pH, moisture, protein, fat, dietary fiber, and carbohydrate contents) of hamburger patties (*p* < 0.05). As the amount of DTP flour increased, the protein, ash, dietary fiber, and carbohydrate content increased, while fat content decreased. The fat contents ranged from 26.92% to 38.43%, and significant differences were observed among the hamburger samples (*p* < 0.05). 

The highest moisture content was observed in the control sample (54.56%), whereas the lowest value was found in the sample enriched with 4% DTP flour (50.47%). It was observed that the dry matter content of the final product increased with an increasing amount of DTP flour (*p* < 0.05). The fat contents were in the range of 34.23% and 26.92% in DTP flour-containing samples. These significant differences in the composition of meatballs were directly related to the composition of DTP flour and its addition to meatballs. It was also reported by various researchers that meatballs supplemented with food ingredients are influenced by these components [30,42,45,58]. On the other side, the addition of DTP flour did not change energy values (*p* > 0.05).

It was determined that the DTP flours used in this study were rich in fiber and approximately 70% of the DTP consisted of dietary fibers (Table 1). These findings are also consistent with the literature, as the primary constituents of tomato peel are pectin, cellulose, hemicelluloses, lignins, and gums [24,59]. In this study, the dietary fiber content of hamburger patties varied between 4.23 and 8.55% dry matter. DTP4 had the significantly highest dietary fiber content among all the treatments (*p* < 0.05). The results indicate that a higher amount of fiber in the peel also results in a higher amount of fiber in the product into which it is incorporated. Similar results were also reported in different studies [30,60,61,62].

pH is a key quality parameter in meat products, impacting their texture, cooking losses, tenderness, and susceptibility to microbial activity [35]. The pH values of hamburger patties were affected by the addition of DTP flour due to its lower pH value (4.18 as given in Table 2) (*p* < 0.05). The hamburger patty samples had a pH value ranging from 5.47 to 5.70. The highest value was observed in the control group, and the lowest pH value was observed in the samples including 4% DTP flour (*p* < 0.05). The reduction in pH value of hamburger patties including DTP flour may be attributed to the presence of tartaric acid and citric acid, the predominant organic acids in tomatoes [35]. These acids could have imparted acidity to the DTP flour utilized in this study. This was also reported by Candogan, who found that the lowest pH value was obtained with the highest inclusion (15%) of tomato paste in beef patties [25]. The water activity values were in the range of 0.881 and 0.890, and no statistically significant difference was found between the patty samples including different amounts of DTP flour (*p* > 0.05). 

### 3.3. TBARS Values of Hamburger Patty Samples

The TBARS value is a significant indicator of lipid oxidation in meat and meat products, reflecting the number of secondary oxidation products such as malondialdehyde (MDA) [63,64]. Table 4 presents the TBARS values of raw and cooked hamburger patty samples. Initial TBARS values for raw hamburger patties ranged from 0.07 to 0.25 mg MA kg^−1^, while those for cooked hamburger patties varied between 0.83 and 2.05 mg MA kg^−1^, respectively. As seen in Table 4, TBARS values decreased with the increase in DTP flour in both raw and cooked hamburger patties (*p* < 0.05). Depending on the heat treatment application, TBARS values increased in all groups (*p*< 0.05). Similar to our results, in a study conducted on chicken breast and beef loin samples that were cooked to an internal temperature of 75 °C, the TBARS values were found to increase with heat treatment [65]. In another study, chicken and duck meats were cooked to internal temperatures of 60, 70, and 80 °C, and an increase in TBARS values was observed in all samples due to the heat effect [66]. 

In both raw and cooked samples, the highest TBARS value was found in the control, while the lowest TBARS values were observed in the DTP3 and DTP4 groups containing high concentrations of DTP flour. Our findings demonstrated that the addition of DTP flour to hamburger patty samples was highly effective in mitigating lipid oxidation. In many studies, it has been shown that tomato peels have a high content of antioxidants, including phenolic compounds and lycopene [27,51,67]. Lycopene, a carotenoid, is the most abundant compound in tomatoes and has been reported to exhibit high antioxidant activity. It acts both indirectly by binding Fe^2+^ and directly by scavenging reactive oxygen species [68]. The antioxidant activity of tomatoes is not solely attributed to lycopene but rather to the synergistic effect of various phenolic compounds [69]. This outcome underscores the antioxidant properties of phenolic compounds, which exhibit high antioxidant activity through chelation of Fe^2+^ and direct scavenging of reactive oxygen species [70,71,72].

### 3.4. Cooking Characteristics of Hamburger Patty Samples

The appearance characteristics of meat products after cooking are an important quality parameter that influences consumer preferences. Particularly, the addition of new ingredients to product formulations can lead to significant shape changes in meatballs. Therefore, maintaining dimensional stability and investigating the effects of the added new components are crucial [73]. For these reasons, in this study, parameters such as water holding capacity (WHC) and cooking efficiency were also analyzed to evaluate the cooking properties and functionality of hamburger patties, and the relevant results are presented in Table 5.

It was found that the cooking yield of hamburger meatballs varied between 69.13% and 80.99%, with the lowest cooking loss observed in the samples with 4% DTP flour added (*p* < 0.05). In addition, the diameter reduction and thickness increase measured in hamburger patty samples varied between 8.14–5.85% and 7.62–15.93%, respectively. (*p* < 0.05). The changes in these parameters were found to be statistically significant (*p* < 0.05). The highest thickness increase and the lowest reduction in diameter were detected in hamburger patties containing 4% DTP flour. The high cooking yield of hamburger patties to which DTP flour was added could be attributed to the high water and oil absorption capacity of DTP flour (Table 1). This can be explained by the decrease in fat content and higher dietary fiber content in samples with increasing DTP flour addition [74,75]. Similarly, in meatball samples containing between 3% and 9% acorn flour, the researchers found that cooking yield increased depending on the addition of acorns [45]. Additionally, some researchers have stated that there is a correlation between dietary fiber addition to meat products, cooking loss, and texture, which has not yet been elucidated [76]. 

In the study, the shrinkage parameter was also determined to observe the changes resulting from protein denaturation and the cooking process [77]. Although the shrinkage parameter increased gradually in samples containing DTP compared to the control group, this difference was not statistically significant (*p* > 0.05). On the other hand, in the study conducted with pork burgers enriched with albedo fiber powder (2.5% and 5%) obtained from yellow passion fruit, it was observed that the shrinkage values were lower than the samples containing 0% albedo powder [78]. 

Moisture and fat retention in hamburger patties are important parameters that determine the sensory properties of the cooked product and therefore the eating quality of the food [45]. Moisture retention values for the hamburger patty samples ranged from 58.57% to 70.73%, while fat retention values were in the range of 74.66% and 79.22%. In hamburger patties, the lowest moisture and oil retention values were in the control group, and the highest moisture and oil retention values were in the group containing 4% DTP (*p* < 0.05). This was because tomato peels are an ingredient rich in dietary fiber, which possess a certain amount of WAC and OAC, as observed in Table 2. 

This situation is associated with the high water and fat retention capacity of many hydroxyl (OH) groups in the structure of dietary fibers [79]. These results are in agreement with Essa and Elsebaie who reported that there was an increment in the water and fat retention values when the levels of date pit powder were increased in beef burgers [80]. Additionally, in another study, it was found that moisture and fat retention rates increased with the addition of purple eggplant flour to meatball samples [79]. 

Various levels of DTP addition significantly influenced the WHC of the patty samples. This characteristic is very important as it affects yield, as well as the juiciness, texture, and flavor of the product, which are characteristics valued by consumers [40,81]. It was found that WHC values tended to increase in all samples with increasing amounts of DTP flour (*p* < 0.05). Similarly, in a study conducted on hamburger patties including a varying range of grape skin flour (0.5% to 3%), WHC gradually increased as well [40]. 

### 3.5. Color Characteristics of Hamburger Patty Samples

**Figure 2:**
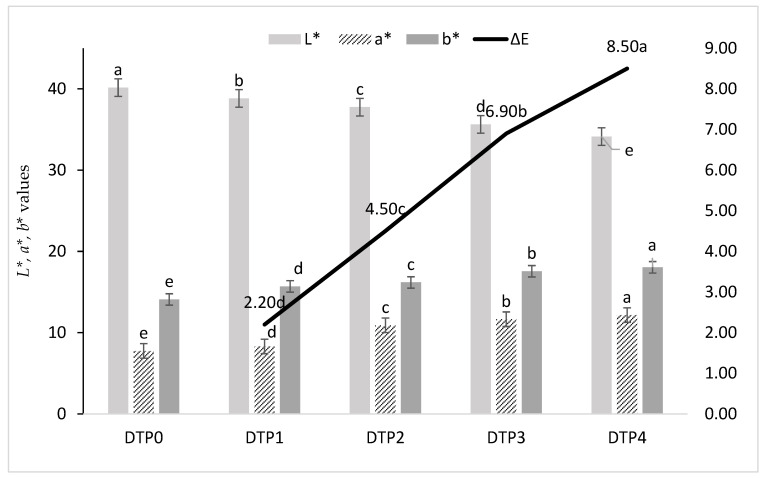
CIE L*, a*, b* color values in hamburger samples and ΔE calculation: DTP0: Control (without DTP flour), DTP1: Burger including 1% DTP flour, DTP2: Burger including 2% DTP flour, DTP3: Burger including 3% DTP flour, DTP4: Burger including 4% DTP flour. ^a–e:^ Means followed by different lower case letters for each color value are significantly different. (*p* < 0.05).

Figure 2 presents the color measurement results of cooked meatball samples. The L* value of the control hamburger patty was 40.15, while it gradually decreased to the range of 38.82–34.12 as the enrichment amount of DTP flour increased in hamburger patties (*p* < 0.05). The a* value of the control group was found to be 7.75, and this measurement also increased with the increasing amount of DTP flour in hamburger samples, which could be an indicator for a more reddish color in patties including DTP flour compared to the control group (*p* < 0.05). This could be attributed to the reddish color of the tomato powder. While the b* value of the control group was measured as 14.09, the meatball samples enriched with DTP flour, which exhibited a more yellowish color, ranged from 15.69 to 18.09 (*p* < 0.05). The total color difference (ΔE) between DTP-added hamburger patties and the control group was highest in DTP4 (8.50), followed by DTP3, DTP2, and DTP1 (*p* < 0.05). Our color findings in hamburger patties showed that the addition of DTP significantly affected the color parameters, and the ΔE values of hamburger patties containing DTP exhibited a significant color change compared to DTP0. Similar to our results, the addition of different concentrations of pumpkin flour into hamburger patties significantly differed the color parameters [82]. Similar color findings have also been reported in tomato pomace cookies [8] and biscuits with tomato peel flour [56]. In summary, it has been concluded that the addition of DTP to meat products significantly affects the color parameters (*p* < 0.05). Based on these findings, the use of DTP in meat products can enhance color quality, and the potential use of this component should be evaluated in further studies.

### 3.6. Textural Properties of Hamburger Patties

In this study, the addition of DTP was found to affect all textural parameters analyzed (*p* < 0.05) (Table 6). As the amount of DTP flour increased in the hamburger patty samples, the hardness and chewiness parameters gradually increased (*p* < 0.05). Other analyzed textural parameters (gumminess, cohesiveness, and springiness) were found to decrease with the addition of DTP, and there were significant differences compared to the control group (*p* < 0.05). Particularly, the cohesiveness index of meatballs containing DTP flour was significantly different in the DTP2, DTP3, and DTP4 groups, as compared to DTP0 (*p* < 0.05). 

Factors such as stromal protein content, extracted protein, degree of comminution, and the type of ingredients added to meat products greatly affect the textural properties of meat and meat products. In addition, ingredients such as extenders, binders, and starch added to hamburger patties play an important role in determining the hardness of the product. Therefore, using higher amounts of DTP in this study resulted in greater firmness and chewiness, especially in DTP3 and DTP4 patties, which contain high dietary fiber. Because, as is known, most of the fiber content of DTP consists of cellulose and lignin [83,84]. Specifically, pectin content affects springiness, while cellulose and lignin content influence hardness and gumminess. Moreover, similar to how the fiber content of DTP flour influences the textural properties of hamburger patties, it is recognized that proteins, fats, and other components play a role in shaping the textural properties of the meat products by influencing their water-binding capacity and lipid crystallization states [85,86]. Similar to our study, it was observed that the addition of adzuki bean flour, which has a high carbohydrate content, to hamburger patties increased the hardness and chewiness parameters [58]. Similar textural results have also been obtained by many researchers [11,63,85,87]. 

### 3.7. Sensory Properties of the Hamburger Patty Samples

**Figure 3:**
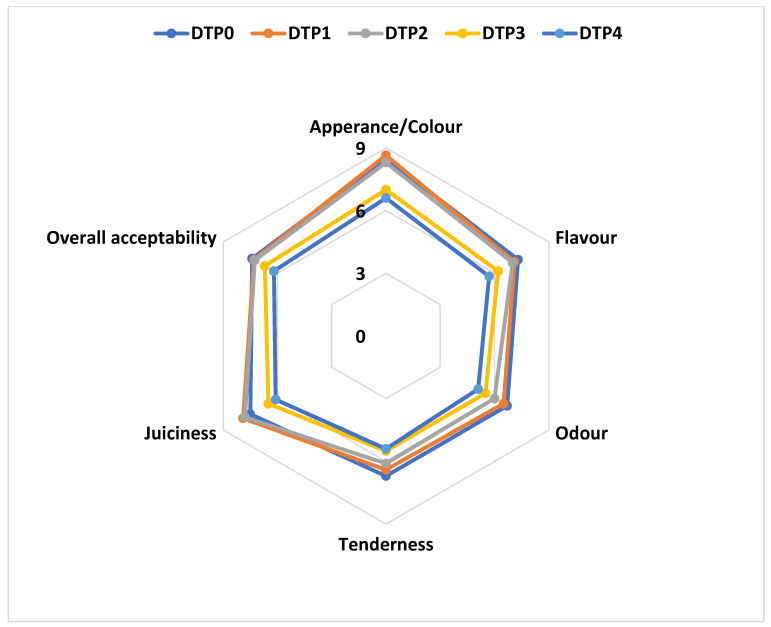
Sensory evaluation results of the hamburger patties: DTP0: Control (without DTP flour), DTP1: Burger including 1% DTP flour, DTP2: Burger including 2% DTP flour, DTP3: Burger including 3% DTP flour, DTP4: Burger including 4% DTP flour.

The sensory evaluation results of the hamburger patty samples including DTP flour are shown in Figure 3. In the study, all sensory parameters were impacted by the addition of DTP flour (*p* < 0.05). In particular, the DTP3 and DTP4 groups differed from the control group, although there was no statistically significant difference between them (*p* > 0.05). The addition of DTP flour enhanced the meatballs’ flavor and odor while also improving their appearance and structure. The DTP2 had higher scores for overall acceptability, taste, texture, and appearance than the control group (*p* > 0.05). However, the DTP4 had lower scores for many evaluated parameters. 

The appearance/color and odor scores for the DTP3 and DTP4 groups were significantly lower than those for the control or the other two DTP-including groups (*p* < 0.05). Groups with high concentrations of DTP, which exhibit a reddish-orange hue when lycopene content rises, may be to blame for the decline in appearance and color liking. An increase in this hue results in a color that is quite different from what is expected in traditional hamburger patties. The results obtained from the tenderness and juiciness parameters were consistent with those measured by instrumental analysis. These two measurements were significantly lower in the DTP4 group. The addition of DTP flour had a negative effect on the indices of flavor and overall acceptability, particularly at concentrations of 3% and 4%. The tomato flavor in the hamburger patties with DTP caused alterations in taste, deviating from the typical flavor associated with this meat product.

Consequently, it is possible to argue that adding DTP flour to hamburger patties did not adversely affect the samples’ sensory qualities. When compared to the control group, the overall acceptability parameter of hamburger patties including 1% or 2% DTP flour had higher scores than the control and graded with very close scores with the samples including 3% and 4% DTP flour. These sensory findings demonstrated that the addition of DTP flour into hamburger patties up to 2% is acceptable to consumers. 

### 3.8. Principal Component Analysis

**Figure 4:**
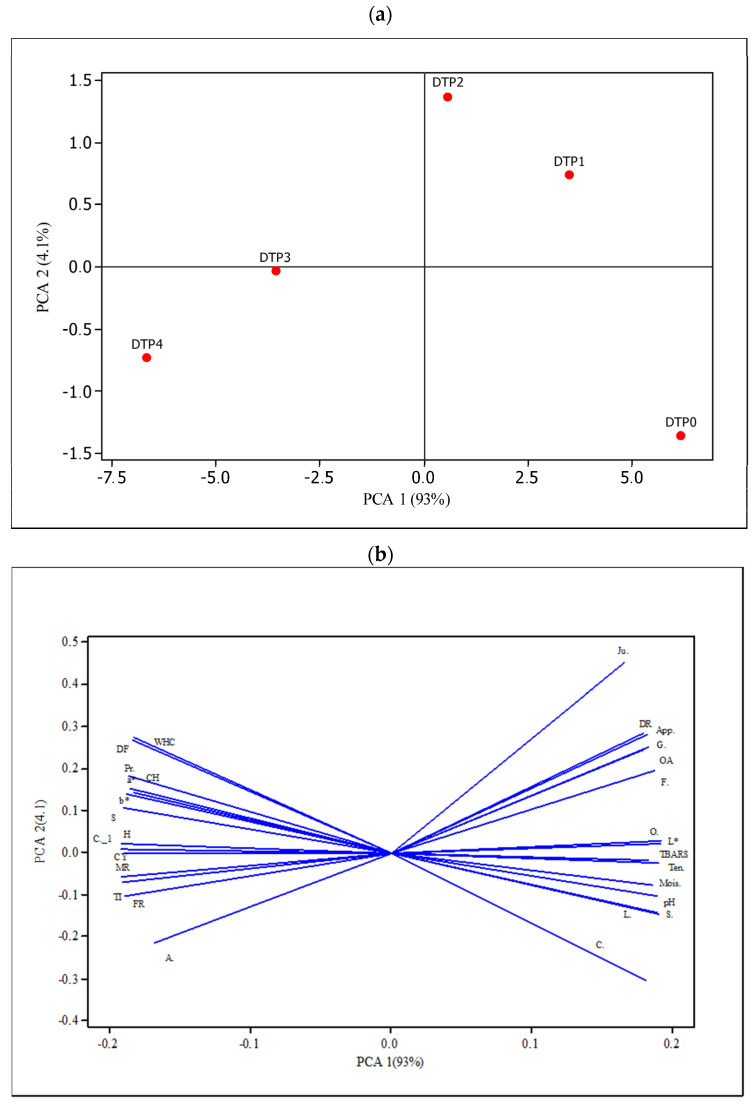
Principle component analysis (PCA) of hamburger patty samples: (**a**) the location of different groups; (**b**) the location of quality parameters; DTP0: Control (without DTP flour), DTP1: Burger including 1% DTP flour, DTP2: Burger including 2% DTP flour, DTP3: Burger including 3% DTP flour, DTP4: Burger including 4% DTP flour.

Physical, chemical, and sensory parameters of DTP-added hamburger patties and relationships among groups with different concentrations of DTP flour were assessed using Principal Component Analysis (PCA). PCA plots help us understand the correlations, similarities, and differences between groups following the addition of DTP to hamburger patties. The arrangement of the principal components is determined by how much of the explained variance in the variables they encompass. Table 7 indicates that the first and second components, representing proportions of 0.930 and 0.041, respectively, had the highest explained variances among all 29 extracted components. As can also be seen in Table 7, most of the information of the dataset is in the first component. The positions of hamburger patty groups are shown in Figure 4a, while the distribution of quality parameters in the space defined by the first and second PCA dimensions is illustrated in Figure 4b. The sum of PC1 and PC2 principal components explains 97.1% of the variation among the hamburger patty groups. PC1 accounts for the majority of the variability (97.1%), while 4.1% is attributed to PC2. It is clearly evident from the results (Figure 4a) that the addition of DTP flour to hamburger patties caused changes in each parameter examined.

It is clearly evident from Figure 4a that the group without the addition of DTP flour, the DTP0 group was the only sample located in the third quadrant. This indicates a change in the parameter values measured in hamburger patties after the addition of DTP flour. Samples DTP1 and DTP2 were located in the first quadrant, while DTP3 and DTP4 samples were in the fourth quadrant, suggesting the least significant difference between these groups. Figure 4b demonstrates that each measured parameter significantly affected the groups with different concentrations of DTP flour (DTP1, DTP2, DTP3, DTP4). As can be seen from Figure 4b, most of the data are associated with the positive part of PC1. All sensory parameters evaluated in hamburger patties, including chewiness, gumminess, and springiness, along with some physicochemical properties, are located in the positive part of PC1. In addition, Figure 4b shows that the DTP1 and DTP2 sample groups are located close to each other in the positive parts of both PCs, indicating a positive correlation between the measurements. This reveals the effect of DTP addition on the samples. Although there were no exact expectations of sample classification before the analysis, the plot of the PCA-1 shows some clustering of hamburger patty samples. In summary, Figure 4b shows that the DTP 2 sample significantly affected the measured parameters. 

### 3.9. Pearson Correlation Results

**Figure 5:**
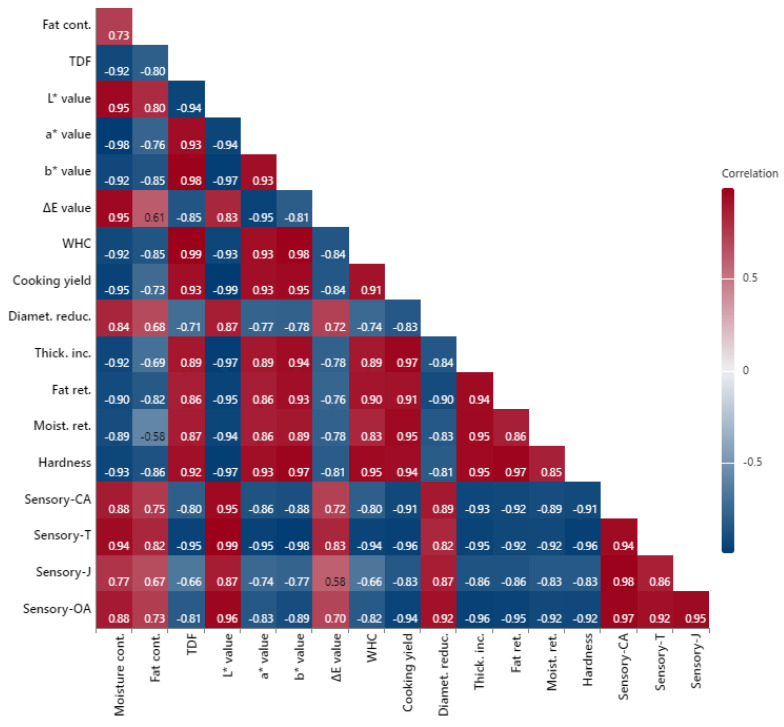
Pearson correlation analysis results. Cont: content, TDF: Total dietary fiber, WHC: Water holding capacity, Ret: retention, CA: color/appearance, T: Tenderness, J: Juiceness, OA: Overall acceptability.

The correlation within some of the dependent variables was evaluated and is demonstrated in Figure 5. Most of the correlations (except three of them; fat content and ΔE, juiciness and ΔE, fat content and moisture retention) were found to be statistically significant (*p* < 0.05). A strong correlation was determined between moisture content and L* value (0.95) or tenderness (0.94). The increase in TDF was significantly correlated with the increase in WHC (0.99), cooking yield (0.93), and hardness (0.92). The correlation value between WHC and hardness was 0.95, which was explained as an increase in WHC resulting in a hard texture. In a similar way, hardness increased with the increasing percentage of fat retention (0.97) or cooking yield (0.94), which was supported by a strong correlation between these parameters. 

On the other hand, statistical evaluation demonstrated negative strong correlations between some of the dependent variables. For instance, an increase in moisture content was negatively correlated with a decrease in TDF (−0.92), WHC (−0.92), cooking yield (−0.95), moisture retention (−0.89), and hardness (−0.93). Additionally, a significant negative correlation value was calculated between tenderness and TDF (0.95) or WHC (0.94). These results could be given as an explanation for the reduction in tenderness with the increasing amount of TDF or WHC as the amount of DTP flour increased in hamburger patties. Similarly, a strong negative correlation was found between all sensory parameters and cooking yield, hardness, moisture, or fat retention values.

## 4. Conclusions

In this study, the potential use of tomato peels as a valuable food ingredient in hamburger patties was investigated, aiming to develop a meat product rich in dietary fiber. The addition of DTP flour improved the cooking properties of hamburger patties, resulting in higher moisture and fat retention and a significant reduction in cooking loss. Overall, our findings indicate that the addition of up to 2 g/100 g of tomato peel flour can enhance the nutritional, textural, technological, and sensory qualities of the hamburger patties without adversely affecting sensory acceptance. Our results provide valuable insights into the potential use of tomato waste as a food ingredient, which potentially provides environmental sustainability by reducing the waste. However, further research is needed to clarify the impact of using tomato peels as an ingredient in different kinds of food products in terms of consumer acceptance, its health effects, food quality, and safety.

### Implications for Research and Practice

This study was conducted using a specific variety of tomato peel flour only in a hamburger patty formulation. Future research should be conducted on the use of different varieties of tomato wastes in different kinds of food products in order to evaluate the effect of this valorization on food quality, safety, consumer acceptance, and economic feasibility. Future studies in this scope will help us gain a more comprehensive understanding of the usability of tomato wastes in food production. Additionally, more detailed studies on the environmental impacts and a cost–benefit analysis of using tomato waste in the food industry should be performed. However, it should not be forgotten that the limitation of this kind of study is that a high concentration of these wastes may lead to unacceptable results in color, sensory, and texture parameters.

## Figures and Tables

**Table 1:** Formulation of hamburger patties (%).

	DTP0	DTP1	DTP2	DTP3	DTP4 *
Ingredients					
Beef	75	74	73	72	71
Bread crumbs	10	10	10	10	10
Water	7	7	7	7	7
Salt	2	2	2	2	2
Black pepper	2	2	2	2	2
Onion powder	2	2	2	2	2
Garlic powder	2	2	2	2	2
DTP flour	0	1	2	3	4

* DTP0: Control (without DTP flour), DTP1: Burger including 1% DTP flour, DTP2: Burger including 2% DTP flour, DTP3: Burger including 3% DTP flour, DTP4: Burger including 4% DTP flour.

**Table 2:** The physicochemical properties of DTP flour (*n* = 2).

Parameters	Mean ± SEM
Moisture (%)	6.20 ± 0.51
aw	0.41 ± 0.80
pH	4.18 ± 0.55
Titratable acidity (%)	7.75 ± 0.86
Protein (%)	10.15 ± 1.19
Fat (%)	1.61 ± 1.51
Ash (%)	5.46 ± 0.65
Total dietary fiber (%) (TDF)	72.88 ± 0.56
Soluble dietary fiber (%) (SDF)	12.13 ± 0.98
Insoluble dietary fiber (%) (IDF)	60.75 ± 0.79
Carbohydrate (%)	76.58 ± 1.09
Oil absorption capacity (%) (OAC)	82.01 ± 0.98
Water absorption capacity (%) (WAC)	50.75 ± 0.73
Swelling capacity (%) (SC)	140.00 ± 0.81

**Table 3:** Compositional, physical, and chemical parameters of cooked hamburger patties formulated with different levels of DTP flour (*n* = 2).

Parameters	DTP0	DTP1	DTP2	DTP3	DTP4 *
pH	5.70 ± 0.01 ^a^	5.62 ± 0.06 ^b^	5.59 ± 0.06 ^b^	5.49 ± 0.03 ^c^	5.47 ± 0.02 ^c^
Water activity (a_w_)	0.889 ± 0.006 ^a^	0.882 ± 0.005 ^a^	0.890 ± 0.008 ^a^	0.885 ± 0.003 ^a^	0.881 ± 0.001 ^a^
Moisture (%)	54.66 ± 1.36 ^a^	54.27 ± 0.92 ^ab^	52.10 ± 0.10 ^bc^	51.79 ± 0.015 ^bc^	50.47 ± 0.50 ^c^
Protein (%DM) ^#^	42.94 ± 0.62 ^d^	44.44 ± 0.64 ^a^	43.67 ± 0.11 ^c^	43.83 ± 0.81 ^bc^	44.10 ± 0.03 ^ab^
Fat (%DM)	38.43 ± 0.92 ^a^	34.23 ± 1.15 ^ab^	32.55 ± 1.04 ^ab^	27.21 ± 2.63 ^b^	26.92 ± 3.47 ^b^
Ash (%DM)	4.57 ± 0.79 ^a^	4.62 ± 0.05 ^a^	5.15 ± 0.04 ^a^	5.17 ± 0.17 ^a^	5.60 ± 0.13 ^a^
Carbohydrate (%DM)	5.61 ± 0.11 ^e^	6.78 ± 0.03 ^d^	9.68 ± 0.02 ^c^	10.41 ± 0.01 ^b^	11.22 ± 0.11 ^a^
Dietary fiber (%DM)	0.07 ± 0.04 ^e^	4.23 ± 0.04 ^d^	5.98 ± 0.03 ^c^	7.77 ± 0.04 ^b^	8.55 ± 0.10 ^a^
Energy value (kcal/100 g)	263.19 ± 4.29 ^a^	261.97 ± 3.56 ^a^	264.41 ± 4.60 ^a^	262.68 ± 12.26 ^a^	263.00 ± 19.20 ^a^

^a–e:^ Means followed by different lowercase letters in the same row, are significantly different. * DTP0: Control (without DTP flour), DTP1: Burger including 1% DTP flour, DTP2: Burger including 2% DTP flour, DTP3: Burger including 3% DTP flour, DTP4: Burger including 4% DTP flour. ^#^ DM: Protein, fat, ash, carbohydrate, and dietary fiber results were calculated as g/100 g dry matter (DM).

**Table 4:** TBARS values (mg MDA/kg meat) of hamburger patties formulated with different levels of DTP flour (*n* = 2).

	DTP0	DTP1	DTP2	DTP3	DTP4 *
Raw	0.23 ± 0.13 ^ay^	0.25 ± 0.13 ^ay^	0.10 ± 0.01 ^ay^	0.08 ± 0.02 ^ay^	0.07 ± 0.01 ^ay^
Cooked	2.05 ± 0.07 ^ax^	1.51 ± 0.03 ^bx^	1.62 ± 0.02 ^bx^	0.93 ± 0.03 ^cx^	0.83 ± 0.03 ^cx^

^a–c:^ Means with different uppercase letters in the same row are significantly different due to the effect of DTP supplementation (*p* < 0.05). ^x,y^: Means with different lowercase letters in the column are significantly different due to the effect of cooking treatment (*p* < 0.05). * DTP0: Control (without DTP flour), DTP1: Burger including 1% DTP flour, DTP2: Burger including 2% DTP flour, DTP3: Burger including 3% DTP flour, DTP4: Burger including 4% DTP flour.

**Table 5:** Cooking measurements and functional results of hamburger patties formulated with different levels of DTP flour (*n* = 2).

	DTP0	DTP1	DTP2	DTP3	DTP4 *
Cooking properties					
Cooking yield (%)	69.13 ± 1.39 ^d^	71.88 ± 0.40 ^d^	74.24 ± 0.41 ^c^	77.08 ± 0.43 ^b^	80.99 ± 1.23 ^a^
Diameter reduction (%)	8.51 ± 0.54 ^a^	8.68 ± 0.43 ^ab^	8.14 ± 0.26 ^ab^	7.32 ± 0.29 ^ab^	5.85 ± 0.98 ^b^
Thickness increase (%)	7.62 ± 0.55 ^c^	9.29 ± 0.93 ^bc^	10.45 ± 0.55 ^bc^	13.03 ± 0.54 ^ab^	15.93 ± 1.29 ^a^
Shrinkage (%)	12.78 ± 1.05 ^a^	13.52 ± 1.56 ^a^	13.93 ± 0.98 ^a^	14.68 ± 3.35 ^a^	14.98 ±4.44 ^a^
Functional properties					
Moisture retention (%)	58.57 ± 1.05 ^c^	61.01 ± 0.12 ^bc^	62.94 ± 1.79 ^bc^	66.86 ± 2.34 ^ab^	70.73 ± 1.72 ^a^
Fat retention (%)	74.66 ± 0.78 ^d^	75.48 ± 0.69 ^cd^	76.06 ± 0.08 ^c^	77.49 ± 0.71 ^b^	79.22 ± 0.58 ^a^
WHC (%)	70.76 ± 0.63 ^d^	75.80 ± 0.23 ^c^	77.93 ± 0.32 ^b^	79.93 ± 1.28 ^a^	81.43 ± 0.75 ^a^

^a–d^: Means followed by different lowercase letters in the same row, are significantly different (*p* < 0.05). * DTP0: Control (without DTP flour), DTP1: Burger including 1% DTP flour, DTP2: Burger including 2% DTP flour, DTP3: Burger including 3% DTP flour, DTP4: Burger including 4% DTP flour.

**Table 6:** Texture profile analysis results in hamburger patties formulated with different levels of DTP flour (*n* = 2).

	DTP0	DTP1	DTP2	DTP3	DTP4 *
Parameters					
Hardness (N)	6260.3 ± 83.0 ^c^	6708.0 ± 293 ^bc^	7094.0 ± 256 ^b^	7707.0 ± 293 ^a^	8113.3 ± 70.7 ^a^
Cohesiveness (N mm)	0.81 ± 0.020 ^a^	0.76 ± 0.011 ^ab^	0.74 ± 0.01 ^b^	0.72 ± 0.01 ^b^	0.71 ± 0.01 ^b^
Springiness (mm)	0.78 ± 0.02 ^a^	0.76 ± 0.01 ^ab^	0.73 ± 0.01 ^bc^	0.71 ± 0.01 ^cd^	0.70 ± 0.01 ^d^
Gumminess (N)	5216.4 ± 10.5 ^a^	5148.1 ± 42.3 ^a^	5144.0 ± 13.90 ^a^	4386.7 ± 13.20 ^b^	4324.8 ± 72.5 ^b^
Chewiness (N mm)	4030.3 ± 21.6 ^d^	4140.9 ± 18.5 ^d^	4443.6 ± 16.3 ^c^	4586.9 ± 57.9 ^b^	4896.4 ± 14.6 ^a^

^a–d:^ Means followed by different lowercase letters in the same row, are significantly different. * DTP0: Control (without DTP flour), DTP1: Burger including 1% DTP flour, DTP2: Burger including 2% DTP flour, DTP3: Burger including 3% DTP flour, DTP4: Burger including 4% DTP flour.

**Table 7:** The eigenvalues and the proportions of the explained variances for the 4 components.

Component	Eigenvalue	Proportion
1	26.983	0.930
2	1.203	0.041
3	0.586	0.020
4	0.228	0.008

## Data Availability

The original contributions presented in the study are included in the article; further inquiries can be directed to the corresponding author.
